# Clinical Impact of the Histopathological Index and Neuroimaging Features Status in Primary Central Nervous System Diffuse Large B-Cell Lymphoma: A Single-Center Retrospective Analysis of 51 Cases

**DOI:** 10.3389/fonc.2022.769895

**Published:** 2022-07-08

**Authors:** Zhou Qi, Lei Duan, Guoqiang Yuan, Jianli Liu, Jian Li, Guoqiang Li, Yue Yu, Yanlong Xu, Shangxian Ma, Yawen Pan, Yinian Zhang

**Affiliations:** ^1^ Department of Neurosurgery, Key Laboratory of Neurology, Gansu Province, Lanzhou University Second Hospital, Lanzhou, China; ^2^ Department of Medical Imaging, Lanzhou University Second Hospital, Lanzhou, China; ^3^ Neurosurgery center of Zhujiang Hospital of Southern Medical University, Guangzhou, China

**Keywords:** primary central nervous system diffuse large B-cell lymphoma, pathological feature, survival rates, radiological features, molecular marker

## Abstract

Primary central nervous system diffuse large B-cell lymphoma (PCNS-DLBCL) is an uncommon non-Hodgkin lymphoma subtype, and its clinical and pathological characteristics remain unclear. PCNS-DLBCL patient data were retrospectively evaluated to determine clinical and pathological characteristics and prognostic factors. Furthermore, prognoses were calculated by Kaplan–Meier and Cox regression models based on clinical observations. In total, 51 immunocompetent patients were enrolled. The median age was 55 (range, 16–82) years, and the male-to-female ratio was 3:2. Headache (n = 19; 37%) and the frontal lobe (n = 16; 31%) were the most common presenting symptom and location, respectively. The median follow-up was 33 (range, 3–86) months, and the median overall survival (OS) and progression-free survival (PFS) were 18 months [95% confidence interval (CI), 21.2–34.2] and 15 months (95% CI, 16.9–28.7), respectively. Ki-67, cluster of differentiation-3, and deep brain involvement were independent prognostic markers. Moreover, multifocal lesions and deep brain involvement were unfavorable independent prognostic markers for PFS. This study indicates that targeted drug development for adverse prognostic factors is possible and provides guidance for clinical treatment decision-making.

## Introduction

Diffuse large B-cell lymphoma (DLBCL) is the most common lymphoma subtype, accounting for 30%–40% of adult non-Hodgkin lymphoma diagnoses. Primary central nervous system diffuse large B-cell lymphoma (PCNS-DLBCL) is a rare subtype of B-cell lymphoma (BCL), representing <1% of all non-Hodgkin lymphomas (1). Moreover, the World Health Organization (WHO) classifies PCNS-DLBCL as a distinct entity of hematolymphoid tumors. PCNS-DLBCL mainly occurs among the elderly, especially those over the age of 60.

Over previous decades, the incidence of primary central nervous system lymphoma (PCNSL) has increased markedly in immunocompetent patients for unknown reasons, whereas the incidence of human immunodeficiency virus (HIV)-associated PCNSLs has declined, possibly due to the development of highly active antiretroviral therapies (2, 3). Recently, clinical trials with DLBCL patients have explored the prognostic effect and interdependence of the cell-of-origin (COO) classification and Hans algorithm, dual expression of MYC and BCL-2 proteins, and MYC, BCL-2, and BCL-6 translocations (4) using complementary DNA microarrays and immunohistochemical staining with various markers, including a cluster of differentiation (CD)10, BCL-6, and multiple myeloma-1/interferon regulatory factor-4 (MUM-1).

Previous studies have identified two DLBCL subtypes using the Hans algorithm: germinal center B-cell-like (GCB) and non-GCB (5–8). Many studies have also suggested that the clinical features of PCNS-DLBCL are particularly relevant. Thus, this study aimed to confirm the prognostic factors of PCNS-DLBCL by Kaplan–Meier and Cox regression models.

## Methods

### Patients

All patients received surgical treatment between January 2012 and February 2021. Patients with HIV and immunodeficiency diseases were excluded. Clinical data were obtained from the medical records and follow-up, including sex, age, clinical symptoms, symptom duration, tumor location, laboratory reports, operative findings, and adjuvant therapy strategy. The PCNS-DLBCL diagnosis required microscopic and immunohistochemical evidence and was identified by two experienced neuropathologists (CD and LD) with no knowledge of the patient’s clinical status. The same neuropathologists reexamined the tumor samples using the 2007 WHO classification guidelines (8).

### Demographic and Clinical Data

Demographic and clinical data were retrospectively collected from patients’ medical records. The location, number of lesions, tumor size, and deep brain involvement were evaluated using magnetic resonance imaging.

### Histological Reexamination and Immunohistochemical Staining

Tissue specimens were fixed in a 10% buffered formaldehyde solution and embedded in paraffin wax. All specimens were cut into 3-mm-thick sections and stained with hematoxylin and eosin following the standard protocol. Specimens were also stained with periodic acid–Schiff with and without diastase, mucicarmine, and alcian blue (pH 2.5).

Immunohistochemical staining was performed using the Envision technique with monoclonal antibodies against CD20 (1:200), BCL-2 (1:100), BCL-6 (1:100), C-MYC (1:100), Ki-67 (1:100), CD3 (1:100), CD10 (1:100), and MUM-1 (1:100). All antibodies were obtained from Proteintech (Wuhan, China). Appropriate positive and negative controls for each antibody were run in parallel. Two observers jointly evaluated the immunohistochemical reactions that were descriptively evaluated according to the cellular compartment and cellular population expression of protein and used the cutoff values of > 0% and > 20% of neoplastic cells expressing CD20, BCL-2, BCL-6, C-MYC, Ki-67, CD3, CD10, and MUM-1 to determine positivity for these markers. The MIB-1 labeling index was calculated in regions of maximal activity and expressed as the percentage of stained nuclear area.

### Outcome Data

Follow-up data were obtained from patient records or by telephone. Progression-free survival (PFS) and overall survival (OS) were the primary endpoints. The last follow-up day was 8 February 2021. OS was assessed from the first diagnosis date until the date of the last follow-up or death. PFS was calculated from the date of the first diagnosis to the date of progression, relapse, death, or last contact.

### Ethics Committee Approval

The surgical procedures and clinical follow-up were conducted under the guidelines and terms of all relevant local legislation and received approval from the Lanzhou University Second Hospital’s ethics committee (number 2021A-524). All patients signed informed consent before the operation.

### Statistical Analyses

The potential prognostic factors were age, sex, lactate dehydrogenase (LDH) level, tumor size, number of lesions, the extent of resection, histopathology, immunohistochemistry, and survival rates or progression rates and analyzed by the Kaplan–Meier method. All variables with a p-value of <0.10 in the univariate analysis were included in the Cox proportional hazards multivariate analysis, used to further investigate the prognostic factor relationships. The chi-squared test was used to compare the survival rate differences among PCNS-DLBCL patients with and without GCB. Bivariate associations between survival or recurrence and the prognostic factors were tested using the log-rank test. All differences were considered statistically significant at a p-value of <0.05. Statistical analyses were performed using SPSS for Windows (version 18.0; IBM SPSS Statistics, IBM Corporation, Armonk, NY, USA).

## Results

**Supplementary Table S1** and **Table 1** summarize patient clinical information. The study included 30 men and 21 women with a median age of 55.0 (range, 16–82) years; three patients were aged <18 years upon initial diagnosis. The most common presenting symptom was headache (n = 19). Other presenting symptoms included motor symptoms (n = 16), vision loss (n = 12), epilepsy (n = 3), hemiplegia (n =10, aphasia (n = 10), and cognitive symptoms (n = 4). The main postoperative complication was venous thrombosis of the lower extremities.

**Table 1:** Initial presentations of the 51 PCNS-DLBCL patients at diagnosis.

Presentation	n=51 (%)
Headache	19 (37.2%)
Dizziness	17 (33.3%)
Motor symptoms	16 (31.3%)
Visual symptoms	12 (23.5%)
Hemiplegia and aphasia	10 (19.6%)
Incidental	5 (9.8%)
Cognitive symptoms	4 (7.8%)
Epilepsy	4 (7.8%)
Decreased consciousness	3 (5.8%)
Nausea/vomiting	3 (5.8%)

**Figure 1:**
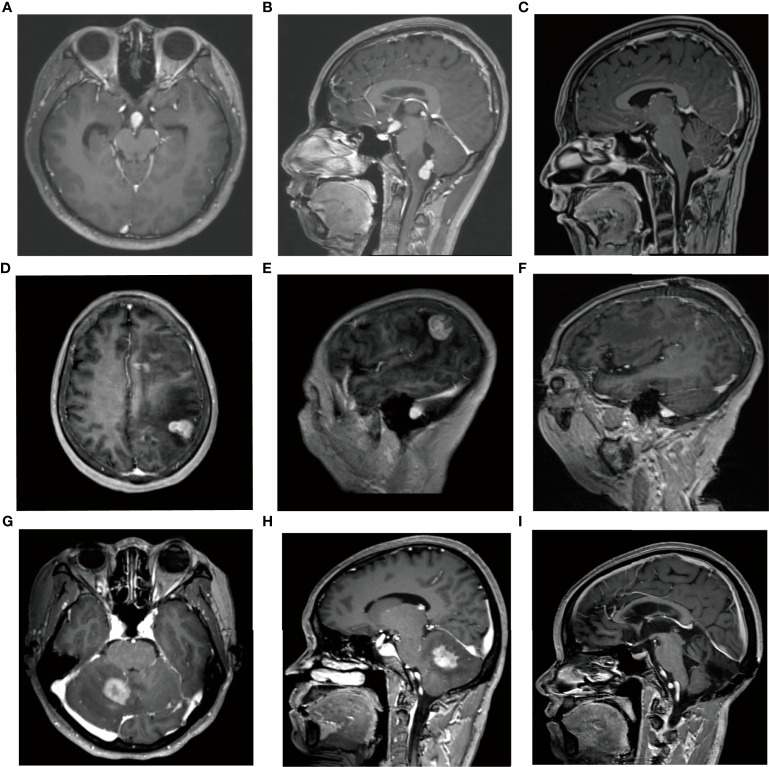
Case 1, male, 16 years old. **(A, B)** MRI-enhanced images showed homogeneous enhancement of tumors in the dorsal side of the brainstem, around the fourth ventricle, and in the pineal region. **(C)** Postoperative MRI showed gross total resection of brainstem lesions. **(D, E)** Case 2, female, 52 years old. Sagittal-enhanced images showed significantly enhanced tumor masses in the left parietal lobe. **(F)** The tumor was totally resected. **(G, H)** Case 38, female, 48 years old, right cerebellar hemisphere aera displayed homogeneous enhancement. **(I)** Postoperative MRI revealed surgery combined with radiotherapy and chemotherapy obtained a good therapeutic effect.

**Figure 2:**
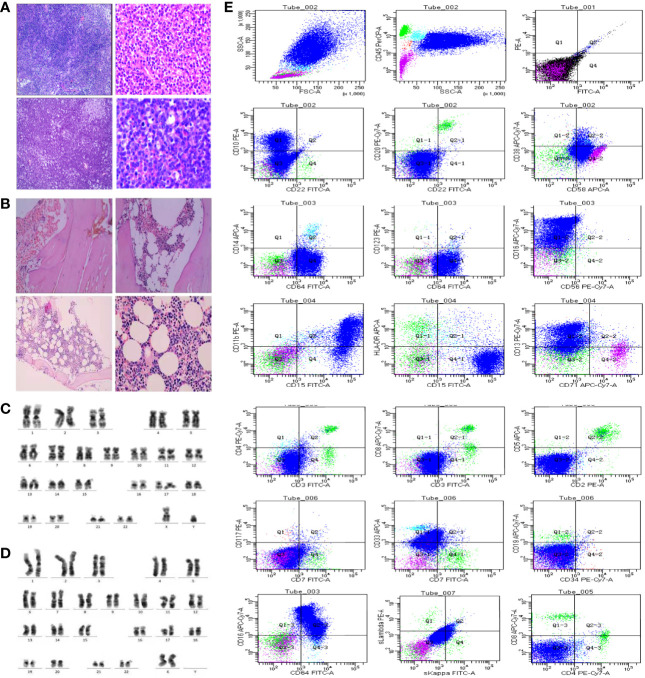
**(A)** HE staining pictures of patients 1 and 2. The above two pictures show patient 1; the bottom two pictures show patient 2 (left: 10×10, right: 10×40). **(B)** Histopathological examination of the bone marrow of patients 1 and 2: the above two pictures showed patient 1; the bottom two picture showed patient 2. **(C, D)** Chromosome karyotype analysis of the two patients. **(E)** Flow cytometry analysis showed that lymphocytes accounted for 9.5% of nuclear cells, and the proportion decreased in patient 1. The proportion of B lymphocytes increased. However, no obvious light chain restriction was found. Primitive region cells account for about 0.5% of nuclear cells.

**Table 2** presents the tumor characteristics. At the initial operation, the tumors occurred in different locations, including the frontal lobe (n = 16), temporal lobe (n = 9), parietal lobe (n = 9), occipital lobe (n = 5), ventricular region (n = 10), basal ganglia and thalamus (n = 4), cerebellopontine angle area (n = 3), cerebellum (n = 3), brainstem (n = 2), and pineal gland (n = 2). Notably, the tumor in case number 47 was in the hypothalamus. There were 17 cases with multifocal lesions and deep brain involvement, involving important structures such as the lateral ventricle, basal ganglia, thalamus, and hippocampus (**Figures 1A–H**). At the same time, we also found that two cases were very special; patient 1 is the youngest reported case of PCNS-DLBCL, and patients 1 and 2 were of great clinical value and significance in analysis, diagnosis, and differential diagnosis of PCNS-DLBCL (**Figures 2A–D**). The tumors ranged from 15 to 150 mm (median, 60 mm) in maximal diameter. In total, 31 (61%) patients underwent gross total resection, and 20 patients (39%) underwent stereotactic biopsy.

**Table 2:** Mass location of the 51 PCNS-DLBCL patients (63 lesions) at diagnosis.

Location	n=63 (%)
Frontal lobe	16 (31.4%)
Parietal lobe	9 (17.6%)
Temporal lobe	9 (17.6%)
Occipital lobe	5 (9.8%)
Ventricular region	10 (19.6%)
Basal ganglia and thalamus	4 (7.8%)
Cerebellopontine angle	3 (5.9%)
Cerebellum	3 (5.9%)
Brainstem	2 (3.9%)
Pineal gland	2 (3.9%)

### Histology and Immunohistochemistry

We obtained specimens from 51 PCNS-DLBCL patients. Histopathological examination revealed tumor tissue composed of lymphoid cells with irregular morphology. The tumor cells displayed abundant eosinophilic cytoplasm, frequent mitoses, and necrotic foci. Most tumor tissues were accompanied by the proliferation of small blood vessels. Furthermore, some lymphoid cells around the blood vessels had apparent proliferation, forming the “cuff” and “starry sky” phenomena. Reed–Sternberg cells were also found in a small number of tumor tissues (**Figure 2A**). All 51 PCNS-DLBCL patients met the 2007 WHO diagnostic criteria (9).

All 51 PCNS-DLBCL patients were identified in the pathology database. All were HIV negative and without an immunosuppressive disease. Furthermore, all patients were strongly positive for CD20 and CD79a, and neoplastic cells expressed pan-B-cell markers, such as CD20, CD79a, and paired box 5 (i.e., PAX5). Seventy percent (36/51) stained positively for MUM-1, 73% (37/51) stained positively for BCL-6, 57% (29/51) stained positively for BCL-2, 31% (16/51) stained positively for CD10, 49% (25/51) stained positively for CD3, and 18% (9/51) stained >90% positive for Ki-67. The Hans algorithm indicated that 32 cases (63%; 32/51) were non-GCB, and 19 (37%; 19/51) were GCB. Finally, 33% (17/51) had BCL-2 and C-MYC coexpression.

### Treatments

All patients had available treatment information. No one received radiotherapy only as an initial treatment because diagnosing PCNS-DLBCL based on only imaging data (i.e., without pathological examination) is very difficult. Moreover, no one received chemotherapy only as an initial treatment or autologous stem cell transplantation. Of those who received postoperative chemotherapy, all received high-dose methotrexate (HD-MTX ≥3.5 g/m2). When their diagnosis was not clear, two patients (4%) received rituximab, and another two patients received treatment options included rituximab and cyclophosphamide, Adria MYC, vincristine, and prednisone (i.e., CHOP)-like regimens as part of their initial therapy, but when their diagnosis was established, HD-MTX therapy was performed.

### Prognostic Factors and Overall Survival

**Figure 3:**
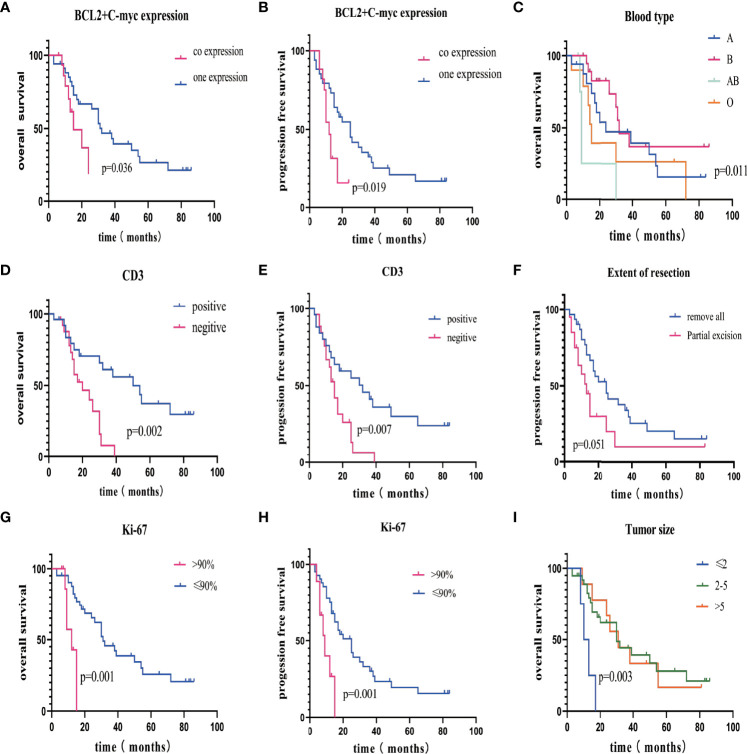
Kaplan–Meier overall survival curve stratified by various risk factors. **(A)** OS in patients with BCL-2 and C-myc coexpression (red line) or one expression (blue line). **(B)** PFS in patients with positive C-myc combined with positive Bcl-2 (red line) or the other conditions (blue line). **(C)** OS in patients with different blood types, there was a significant difference in OS between different blood types. **(D, E)** OS/PFS in patients with positive CD3 (red line) or negative CD3(blue line). **(F)** PFS in patients with GTR (red line) and PR (blue line). **(G, H)** OS/PFS in patients with Ki-67 >90% (red line) or Ki-67 <90% (blue line). **(I)** OS in patients with different tumor size. PFS, progression-free survival; OS, overall survival.

**Figure 4:**
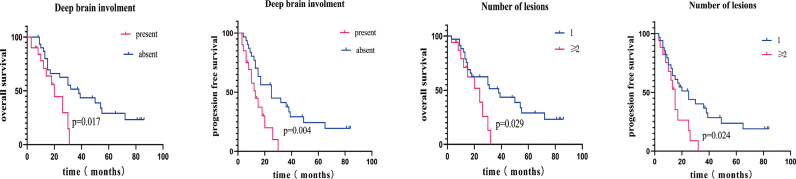
Kaplan–Meier overall survival curve stratified by various risk factors. **(A, B)** OS and PFS in patients with deep brain involvement (red line) or absent deep brain involvement (blue line). **(C, D)** OS/PFS in patients with multifocal lesions (red line) or one lesion (blue line).

All patients had sufficient follow-up data for the analysis. The median follow-up was 33 (range, 3–86) months, and the median OS was 18.0 months [95% confidence interval (CI), 21.2–34.2]. The 1-, 3-, and 5-year OS probability rates were 75% (95% CI, 71–79), 31% (95% CI, 27–35), and 12% (95% CI, 8–15), respectively. **Table 3** summarizes the independent univariate analyses performed for each potential prognostic factor. Ki-67 positivity >90% (p = 0.001), CD3 positivity (p = 0.002), tumor size >5 cm (p = 0.003), less than two lesions (p = 0.029), AB blood type (p = 0.011), BCL-2 and C-MYC coexpression (p = 0.036), and deep brain involvement (p = 0.017) were significantly related to a worse OS (**Figures 3A, C, D, G**, **4A, C**). Patients with gross total resection (GTR) had a greater median OS (32 months; 95% CI, 16.5–47.4) than those with residual tumor after surgery (30 months; 95% CI, 16.2–43.7; p = 0.292). Cox regression analyses revealed that Ki-67 and CD3 were independent prognostic factors for improved OS [Ki-67: p = 0.001, hazard ratio (HR) = 0.135, 95% CI, 0.045–0.411; CD3: p = 0.004, HR = 3.697; 95% CI, 1.145–11.940].

**Table 3:** Univariate and multivariate analyses of risk factors for overall survival.

Prognostic factor		Univariate analysis		Multivariate analysis	
		No (%n=51)	p-value (log-rank test)	p-value (cox regression)	Relative risk (95% CI)
Gender	Female	21 (41.2%)	0.297		
	Male	30 (58.8%)			
Age	≥60	15 (29.4%)	0.362		
	<60	36 (70.6%)			
Ki-67	≥90%	9 (17.6%)	<0.001	0.001	0.176 (0.061–0.505)
	<90%	42 (82.4%)			
Bcl-2	Positive	29 (56.8%)	0.538		
	Negative	22 (43.2%)			
C-myc	Positive	28 (54.9%)	0.203		
	Negative	23 (45.1%)			
Bcl-6	Positive	37 (72.5%)	0.456		
	Negative	14 (27.5%)			
MUM-1	Positive	36 (70.5%)	0.292		
	Negative	15 (29.5%)			
CD3	Positive	25 (49.1%)	0.002	0.004	3.490 (1.498–8.831)
	Negative	26 (50.9%)			
CD10	Positive	16 (31.3%)	0.817		
	Negative	35 (68.7%)			
HANS	GCB	19 (37.2%)	0.955		
	NGCB	32 (62.8%)			
Size	≤2cm	4 (7.8%)	0.003	0.069	0.356 (0.139–0.910)
	2–5cm	38 (74.5%)			
	>5cm	9 (17.7%)			
Number of lesions	1	34 (66.6%)	0.029	0.255	1.496 (0.449–4.981)
	≥2	17 (33.4%)			
EOR	GTR	31 (60.7%)	0.391	0.705	1.377 (0.458–4.135)
	PR	20 (39.3%)			
Blood type	A+	17 (33.3%)	0.011	0.298	1.071 (0.721–1.592)
	B+	19 (37.2%)			
	AB+	5 (9.8%)			
	O+	10 (19.7%)			
LDH	≤215U/L	36 (70.5%)	0.839		
	>215U/L	36 (29.5%)			
BCL-2 and C-myc	co-expression	17 (33.3%)	0.036	0.626	0.642 (0.108–3.816)
	one-expression	34 (66.6%)			
KPS score	>70	31(60.8%)	0.988		
	≤70	20(39.2%)			
Deep brain involment	present	20 (39.2%)	0.017	0.090	0.560 (0.152–2.061)
	absent	31 (60.8%)			

GCB, germinal center B cell; LDH, lactate dehydrogenase, EOR, extent of resection; GTR, gross total resection; PR, partial resection.

### Prognostic Factors and Progression-Free Survival

At the final follow-up, 37 patients experienced progression, defined as aggravation, recurrence, and death. The median PFS was 17.0 months (95% CI, 13.6–20.4). Progression occurred in 18 of 30 male PCNS-DLBCL patients and 18 of 21 female PCNS-DLBCL patients. Men with PCNS-DLBCL had an 18.7% 5-year PFS rate, and women had a rate of 6.8%, but log-rank tests indicated no association between sex and PFS (p = 0.167). Progression occurred in 26 of 36 PCNS-DLBCL patients younger than 60 and 11 of 15 PCNS-DLBCL patients over 60. Patients younger than 60 had a 14.4% 5-year PFS rate, and patients older than 60 had a 22.5% rate, but age and PFS were also not associated (log-rank test; p = 0.281).

Progression occurred in 7 of 9 (77.7%) PCNS-DLBCL patients with Ki-67 positivity >90% and 12 of 42 (28.5%) with Ki-67 positivity <90%. Those with Ki-67 positivity >90% had a 0% 5-year PFS rate, and those with Ki-67 positivity <90% had a 19.5% rate. Log-rank tests indicated a significant relationship between Ki-67 and PFS (p = 0.001).

Progression occurred in 8 of 25 (32%) PCNS-DLBCL patients with CD3 positivity and 6 of 26 (23%) with CD3 negativity. CD3-positive patients had a 30% 5-year PFS rate, but CD3-negative patients had a 0% rate; CD3 and PFS were significantly associated (log-rank test; p = 0.007).

Progression occurred in 10 of 34 (29.4%) PCNS-DLBCL patients with less than two lesions and 4 of 17 patients (23.5%) with more than two lesions. Patients with less than two lesions had a 19% 5-year PFS rate, and patients with more than two lesions had a 0% rate; the lesion number and PFS were significantly associated (log-rank test; p = 0.024).

Progression occurred in 8 of 31 (25.8%) PCNS-DLBCL patients receiving GTR and 6 of 20 (30.0%) patients receiving partial excision. Patients with GTR had a 20.3% 5-year PFS rate, and patients receiving partial excision had a 5% rate. Importantly, PCNS-DLBCL patients with BCL-2 and C-MYC coexpression had a 0% 5-year PFS rate, whereas patients with BCL or C-MYC expression had a 20% rate. Ki-67 positivity >90% (p = 0.001), CD3 positivity (p = 0.007), two or more lesions (p = 0.024), BCL-2 and C-MYC coexpression (p = 0.019), partial excision (p = 0.051), and deep brain involvement (p = 0.004) were significantly associated with worse PFS (**Figures 3B, E, F, H**, **4B, D**). **Table 4** summarizes the other factors.

**Table 4:** Univariate and multivariate analyses of risk factors for progression-free survival.

Prognostic factor		Univariate analysis		Multivariate analysis	
		No (%n=51)	p-value (log-rank test)	p-value (Cox regression)	Relative risk (95% CI)
Gender	Female	21 (41.2%)	0.167		
	Male	30 (58.8%)			
Age	≥60 years	15 (29.4%)	0.281		
	<60 years	36 (70.6%)			
Ki-67	≥90%	9 (17.6%)	0.001	0.005	0.256 (0.100–0.657)
	<90%	42 (82.4%)			
Bcl-2	Positive	29 (56.8%)	0.858		
	Negative	22 (43.2%)			
C-myc	Positive	28 (54.9%)	0.187		
	Negative	23 (45.1%)			
Bcl-6	Positive	37 (72.5%)	0.958		
	Negative	14 (27.5%)			
MUM-1	Positive	36 (70.5%)	0.097	0.441	0.925 (0.308–2.782)
	Negative	15 (29.5%)			
CD3	Positive	25 (49.1%)	0.007	0.009	2.591 (0.923–7.271)
	Negative	26 (50.9%)			
CD10	Positive	16 (31.3%)	0.548		
	Negative	35 (68.7%)			
HANS	GCB	19 (37.2%)	0.298		
	NGCB	32 (62.8%)			
Size	≤2cm	4 (7.8%)	0.086	0.442	0.765 (0.312–1.873)
	2~5cm	38 (74.5%)			
	>5cm	9 (17.7%)			
Number of lesions	1	34 (66.6%)	0.024		
	≥2	17 (33.4%)			
EOR	GTR	31 (60.7%)	0.051	0.088	1.926 (0.751–4.942)
	PR	20 (39.3%)			
Blood type	A+	17 (33.3%)	0.283		
	B+	19 (37.2%)			
	AB+	5 (9.8%)			
	0+	10 (19.7%)			
LDH level	≤215U/L	36 (70.5%)	0.929		
	>215U/L	15 (29.5%)			
BCL-2 and C-myc	Coexpression	17 (33.3%)	0.019	0.532	0.620 (0.133–2.898)
	One expression	34 (66.6%)			
KPS score	>70	31(60.8%)	0.543		
	≤70	20(39.2%)			
Deep brain involment	Present	20 (33.3%)	0.004	0.008	0.373 (0.179–0.775)
	Absent	31 (66.7%)			

GCB, germinal center B cell; LDH, lactate dehydrogenase, EOR, extent of resection; GTR, gross total resection; PR, partial resection.

## Discussion

The fifth edition of the WHO Classification of Tumors of the Central Nervous System (CNS), published in 2021, has emphasized molecular diagnostics for CNS tumor classification (10). This study investigated the role of clinicopathological features and molecular diagnostic factors in predicting patient prognosis. We found seven prognostic factors that significantly correlated with OS and six that significantly related to PFS. We also found that BCL-2 and C-MYC coexpression can act as a hazard factor of PCNS-DLBCL, and smaller tumors had shortened survival times. Deep tumor involvement or multifocal lesions were also significantly associated with OS and PFS, and CD3 was related to good PCNS-DLBCL outcomes as a T-cell marker. Together, these results suggest that molecular pathological diagnosis may play a vital role in the prognosis of PCNS-DLBCL patients.

In our study, more PCNS-DLBCL patients were <60 years old, contrary to a previous study (11) that reported a median age at PCNSL diagnosis of 66 years. Occurrence in children is very rare. However, in our series, one patient (1/51) was younger than 18 years old upon initial diagnosis, and the age of 16 was the youngest case of PCNS-DLBCL found in China.

In our study, the smaller the tumor, the shorter the survival time. It is possible that the tumor tissue was deep, which may destroy the deep brain conduction bundle, leading to a considerably shorter survival time and higher recurrence risk. At the same time, deep brain involvement was an independent risk factor in multivariate analysis, but another study reported no difference in deep brain involvement between patients with and without recurrence (12). It is generally believed that PCNS-DLBCL is highly sensitive to chemotherapy and radiotherapy, and surgical treatment is limited to diagnostic biopsies. However, in our study, the extent of resection may correlate with PFS. Technical advances in neurosurgery have increased the safety of PCNSL surgical resections (13, 14). Therefore, we propose reconsidering tumor removal to treat a single lesion amenable to resection.

The GCB-like DLBCL subtype has a better prognosis than the activated B-cell-like subtype (15, 16). It is well known that PCNS-DLBCL has a variety of molecular characteristics, and the primary factors closely related to prognosis are BCL-2/MYC double expression, BCL-2/C-MYC double aberrations (17), and BCL-6 rearrangements, which are often recognized using cytogenetic fluorescence *in situ* hybridization (FISH) studies (18). C-MYC/BCL-2 double-hit lymphoma has been recognized as a chromosomal break involving MYC and BCL-2, which is very rare, representing 3% of all DLBCL cases (19). In our series, 33% (17/51) of patients had C-MYC/BCL-2 coexpression, and coexpression was correlated with OS and PFS. C-MYC and BCL-2 have also been associated with resistance, a poor response to therapy, and a worse survival rate in PCNS-DLBCL patients (16, 20). We also found C-MYC/BCL-2 as a new predictive factor, correlating with survival time and occurrence independent of the COO subtype and Hans algorithm. In our study, 53% of cases had C-MYC expression, which was significantly lower than that reported by Gill et al. (73%) (21) and Brunn et al. (92%) (22). Additionally, the MYC FISH study provided crucial information for differentiating PCNS-DLBCL from other lymphoma subtypes. As a result, FISH staining should be performed as soon as possible to observe chromosomal site mutations (18).

Although some studies determined that MUM-1, CD10, and BCL-6 were favorable prognostic markers (23), other studies and ours did not find a correlation between these factors and prognosis (24, 25). As a T-cell marker, CD3-positive PCNS-DLBCL is very rare. In our cohort study, we found that CD3 positivity was associated with a significantly better median OS than CD3 negativity in univariate and multivariate analyses. Interestingly, blood type was a prognostic factor for OS in the univariate analysis. Thus, we are the first to demonstrate that blood types significantly correlate with the prognosis of PCNS-DLBCL. In our study, the AB blood type was the least represented, and type AB patients had the worst prognosis, which deserves further investigation. Multivariate analysis identified Ki-67 and CD3 as independent prognostic factors for survival time. Moreover, Ki-67, CD3, and deep brain involvement were independent prognostic factors for PFS.

### Limitations

There are some limitations to this study. First, our study was a retrospective study; therefore, potential bias is inevitable. Second, the median follow-up time was not sufficiently long, <3 years in several cases. Third, the postoperative imaging data were not complete. Moreover, the small cohort in this study might not be well suited to an assessment of our treatment policy.

## Conclusion

This retrospective study spanned 9 years and analyzed the demographic and clinicopathological features of PCNS-DLBCL patients.

For OS, CD3 positivity was an independent favorable prognostic factor, and Ki-67 positivity>0%, tumor size <2 cm, multifocal lesions, BCL-2 and C-MYC coexpression, and deep brain involvement were independent adverse prognostic factors.

For PFS, the extent of resection was an important prognostic factor. Therefore, we recommend GTR for a single lesion. Furthermore, Ki-67 positivity>0%, multifocal lesions, BCL-2 and C-MYC coexpression, and deep brain involvement were independent adverse prognostic factors. The diagnosis and differential diagnosis of PCNS-DLBCL are very difficult. Therefore, it is important to diagnose PCNS-DLBCL based on molecular characteristics and FISH staining. Furthermore, early postoperative chemotherapy and radiotherapy may play a vital role in preventing recurrence.

## Data Availability Statement

The original contributions presented in the study are included in the article/**Supplementary Material**. Further inquiries can be directed to the corresponding authors.

## Ethics Statement

The studies involving human participants were reviewed and approved by the Lanzhou University Second Hospital’s ethics committee. Written informed consent to participate in this study was provided by the participants’ legal guardian/next of kin. Written informed consent was obtained from the minor(s)’ legal guardian/next of kin for the publication of any potentially identifiable images or data included in this article.

## Author Contributions

Conception and design: ZQ. Acquisition of data: all authors. Analysis and interpretation of data: ZQ and YZ. Drafting the article: ZQ, LD, GY, JL, YP, and YZ. Critically revising the article: YZ. Reviewed submitted version of manuscript: ZQ and YZ. Approved the final version of the manuscript on behalf of all authors: YZ. Statistical analysis: ZQ. Study supervision: YZ. All authors contributed to the article and approved the submitted version.

## Funding

This work was supported by the National Natural Science Foundation of China (81771297), Research Innovation Group Project of Gansu Province (21JR7RA432), and Gansu Province Health Industry Research Project (GSWSKY2018-01).

## Conflict of Interest

The authors declare that the research was conducted in the absence of any commercial or financial relationships that could be construed as a potential conflict of interest.

## Publisher’s Note

All claims expressed in this article are solely those of the authors and do not necessarily represent those of their affiliated organizations, or those of the publisher, the editors and the reviewers. Any product that may be evaluated in this article, or claim that may be made by its manufacturer, is not guaranteed or endorsed by the publisher.

## Supplementary Material

Supplementary Table S1 Clinicopathological features of 51 primary PCNS-DLBCL cases.

## Abbreviations

PCNS-DLBCL, primary central nervous system diffuse large B-cell lymphoma; OS, overall survival; PFS, progression-free survival, EOR, extent of resection; GTR, gross of resection.
