# Supplementary material for: Clinical Impact of the Histopathological Index and Neuroimaging Features Status in Primary Central Nervous System Diffuse Large B-Cell Lymphoma: A Single-Center Retrospective Analysis of 51 Cases
Source: Front Oncol. 2022 Jul 8;12:769895. doi: 10.3389/fonc.2022.769895 (PMC9304881; doi:10.3389/fonc.2022.769895)
Supplement: Supplementary Table S1 — Clinicopathological features of 51 primary PCNS-DLBCL cases. [file Table_1.docx]

sTable 1 clinicopathological features of 51 primary PCNS-DLBCL cases

| Case | Age | Sex | CD79a | CD20 | KPS Score | LDH level | Size | Cell origin | Time | Status |
| --- | --- | --- | --- | --- | --- | --- | --- | --- | --- | --- |
| 1 | 16 | F | Positive | Positive | 90 | 133 | 2.4*1.3*2.2 | GCB | 7 | Alive |
| 2 | 52 | M | Positive | Negative | 70 | 149 | 2.8*3.2*3.0 | NGCB | 6 | Alive |
| 3 | 27 | F | Positive | Positive | 50 | 214 | 6.3×6.2×5.2 | GCB | 31 | Death |
| 4 | 53 | M | Negative | Positive | 90 | 124 | 2.8×1.2×2.3 | NGCB | 30 | Death |
| 5 | 56 | F | Positive | Positive | 80 | 181 | 0.9×5.3×2.8 | NGCB | 30 | Death |
| 6 | 44 | M | Positive | Positive | 90 | 209 | 2×2×3 | GCB | 3 | Death |
| 7 | 49 | F | Positive | Positive | 80 | 187 | 4.1×3.6×3.2 | NGCB | 18 | Alive |
| 8 | 60 | F | Positive | Positive | 80 | 178 | 1.5×0.9×1.8 | GCB | 17 | Death |
| 9 | 55 | F | Positive | Positive | 80 | 141 | 2.5×3×3 | GCB | 15 | Death |
| 10 | 51 | F | Positive | Positive | 70 | 280 | 4.6×4.7×3.6 | NGCB | 20 | Alive |
| 11 | 43 | M | Positive | Positive | 80 | 194 | 2.8×1.6×0.8 | NGCB | 20 | Death |
| 12 | 55 | F | Positive | Positive | 50 | 145 | 3.4×2.9×2.9 | GCB | 19 | Alive |
| 13 | 53 | M | Positive | Positive | 70 | 576 | 2.4×2.1×2.5 | GCB | 18 | Death |
| 14 | 51 | F | Positive | Positive | 80 | 314 | 6.8×9.6×12.1 | NGCB | 15 | Death |
| 15 | 61 | F | Positive | Positive | 50 | 152 | 4×3×4 | NGCB | 24 | Alive |
| 16 | 48 | M | Positive | Positive | 80 | 197 | 2.3*1.4*3 | NGCB | 15 | Death |
| 17 | 53 | F | Positive | Positive | 80 | 179 | 3.6*3.7*3.5 | NGCB | 13 | Alive |
| 18 | 58 | M | Positive | Positive | 90 | 183 | 2.4*1.4*2.1 | NGCB | 12 | Death |
| 19 | 62 | F | Positive | Positive | 70 | 166 | 1.8*2.1*1.5 | GCB | 13 | Death |
| 20 | 32 | F | Positive | Positive | 90 | 187 | 4.6×4.7×3.6 | GCB | 6 | Alive |
| 21 | 58 | M | Positive | Positive | 90 | 472 | 4.2*1.8*3.5 | NGCB | 9 | Death |
| 22 | 45 | F | Positive | Positive | 80 | 284 | 4*4*5 | NGCB | 8 | Alive |
| 23 | 40 | F | Positive | Positive | 80 | 250 | 3.3*2.8*3.2 | GCB | 14 | Alive |
| 24 | 59 | F | Positive | Positive | 80 | 141 | 3.7*2.5*3 | NGCB | 10 | Death |
| 25 | 44 | F | Positive | Positive | 40 | 306 | 2.3*1.5*0.5 | NGCB | 10 | Alive |
| 26 | 52 | M | Positive | Positive | 40 | 176 | 4.6×4.7×3.6 | NGCB | 10 | Alive |
| 27 | 54 | F | Positive | Positive | 60 | 265 | 1.6*1.9*1.6 | NGCB | 8 | Death |
| 28 | 70 | M | Positive | Negative | 80 | 151 | 2.2*1.2*1.2 | NGCB | 10 | Death |
| 29 | 60 | F | Positive | Positive | 80 | 223 | 5.7*4.4*4.3 | GCB | 48 | Alive |
| 30 | 78 | M | Positive | Positive | 80 | 211 | 2.5*2*2 | NGCB | 48 | Alive |
| 31 | 65 | F | Positive | Positive | 80 | 153 | 3.6*3.9*4.0 | NGCB | 12 | Death |
| 32 | 62 | F | Positive | Positive | 80 | 352 | 15*10*8 | NGCB | 9 | Death |
| 33 | 53 | F | Positive | Positive | 60 | 205 | 2*2*0.2 | NGCB | 39 | Death |
| 34 | 54 | M | Positive | Positive | 70 | 193 | 6.6*5.0*4.2 | NGCB | 38 | Death |
| 35 | 82 | M | Positive | Positive | 60 | 208 | 4*3*4 | GCB | 13 | Death |
| 36 | 59 | M | Positive | Positive | 80 | 277 | 4*4*3 | NGCB | 37 | Alive |
| 37 | 61 | F | Positive | Positive | 90 | 263 | 4*5*6 | NGCB | 55 | Death |
| 38 | 48 | M | Positive | Positive | 50 | 190 | 2.1*2.4*2.3 | NGCB | 54 | Death |
| 39 | 60 | F | Positive | Positive | 80 | 154 | 4*2.4*0.8 | GCB | 50 | Death |
| 40 | 56 | F | Positive | Positive | 80 | 198 | 5*4*1 | GCB | 72 | Death |
| 41 | 59 | M | Positive | Positive | 40 | 155 | 3*4*3 | GCB | 14 | Death |
| 42 | 73 | F | Positive | Positive | 70 | 194 | 3*2*1 | GCB | 32 | Death |
| 43 | 64 | M | Positive | Positive | 80 | 231 | 4*3*4 | NGC B | 30 | Death |
| 44 | 27 | F | Positive | Positive | 90 | 184 | 4*5*4 | GCB | 65 | Alive |
| 45 | 46 | F | Positive | Positive | 90 | 171 | 3*2.1*1 | NGCB | 84 | Alive |
| 46 | 51 | M | Positive | Positive | 40 | 133 | 2.5*2*1 | NGCB | 86 | Alive |
| 47 | 47 | M | Positive | Positive | 60 | 301 | 2.5*2*0.4 | NGCB | 3 | Death |
| 48 | 71 | M | Positive | Positive | 60 | 247 | 2*3*2 | NGCB | 83 | Alive |
| 49 | 73 | F | Positive | Positive | 90 | 265 | 4*5*6 | GCB | 81 | Alive |
| 50 | 51 | M | Positive | Positive | 70 | 167 | 4*6*3 | NGCB | 24 | Death |
| 51 | 36 | F | Positive | Positive | 90 | 107 | 4*6*6 | GCB | 26 | Death |
